# From laboratory to point of care: Clinical performance and validation of bSens.INR for prothrombin time and INR monitoring

**DOI:** 10.1016/j.plabm.2026.e00564

**Published:** 2026-10-27

**Authors:** Julia Konzen Moreira, Gabriela Victória de Mello Jantzch, Duane da Silva Moraes, Bruna Ferri Serafini, Milleny Germann Souza, Marcele Arais Hocevar, Rodrigo Ritzel Bernasconi, Fernanda Santos dos Santos, Willyan Hasenkamp Carreira

**Affiliations:** a Postgraduate Program in Chemistry, Federal University of Rio Grande do Sul, 91501-970, Brazil; b Postgraduate Program in Environmental Quality, Feevale University, 93525-075, Brazil; c Postgraduate Program in Mining, Metallurgical and Materials Engineering, Federal University of Rio Grande do Sul, 91501-970, Brazil; d Postgraduate Program of Collective Health, University of Vale do Rio dos Sinos, 93022-750, Brazil; e Undergraduate Program in Systems Analysis and Development, University of Vale do Rio dos Sinos, 93022-750, Brazil; f Postgraduate Program in Materials Engineering and Technology, Pontifical Catholic University of Rio Grande do Sul, 90619-900, Brazil; g Postgraduate Program in Bioengineering and Biotechnology, Swiss Federal Institute of Technology Lausanne, AI 1206 CH-1015, Switzerland; h Biosens Biosensor Development and Manufacturing Ltda., 93022-715, Brazil

**Keywords:** International normalized ratio, Prothrombin time, Point-of-care, Anticoagulation, Warfarin, Clinical validation, Monitoring

## Abstract

**Background:**

Anticoagulant therapy with vitamin K antagonists, particularly warfarin, requires periodic monitoring of prothrombin time (PT) and the International Normalized Ratio (INR) to maintain patients within the therapeutic range and reduce thromboembolic and hemorrhagic risks. Point-of-care (POC) devices may improve the accessibility and frequency of anticoagulation monitoring.

**Objectives:**

To evaluate the clinical performance of bSens.INR, a POC system for quantitative PT/INR determination, by comparison with a laboratory reference method and assessment of precision and usability.

**Patients/methods:**

Clinical validation included 77 participants and 200 capillary blood determinations performed in duplicate (400 bSens.INR tests), with corresponding venous samples analyzed by laboratory reference methods. Accuracy, repeatability, intermediate precision, and usability were assessed. Potential interfering factors were evaluated descriptively and exploratorily.

**Results:**

Overall, 98,5% of results met the predefined accuracy acceptance criteria, demonstrating adequate agreement with the laboratory reference method. Accuracy decreased at INR values above 3.7. All duplicate measurements met the repeatability acceptance criteria, with variability below 10%. Intermediate precision and usability assessments further supported system performance under the evaluated conditions. Exploratory assessment of potential interfering factors did not establish causal relationships but identified factors warranting further investigation.

**Conclusions:**

bSens.INR demonstrated adequate clinical performance for PT/INR determination under the evaluated conditions. These findings provide clinical evidence supporting its regulatory evaluation by the Brazilian Health Regulatory Agency (ANVISA). Further studies should investigate higher INR ranges, potential interfering factors, and real-world performance, including self-monitoring.

## Highlights

• bSens.INR enables rapid point-of-care PT/INR measurement using fingertip capillary blood.
• The system achieved 98,5% of results within the ISO 17593:2022 accuracy acceptance criteria.
• Repeatability testing showed variability below 10%, supporting consistent duplicate measurements.
• Instrument, test day, strip lot, and replicate had no significant effect on deviations from the reference method (p > 0.05).
• Clinical and usability findings support the potential of bSens.INR for point-of-care PT/INR monitoring and future home self-testing.

## 1 Introduction

Currently, most laboratory diagnostic tests are performed in a centralized setting, following a workflow that includes laboratory test requisition, venous blood collection, sample transportation to the laboratory, test performance, and report issuance. In addition, the completion of this workflow requires complex infrastructure, specific and high-cost equipment, and qualified personnel, as illustrated in Fig. 1. This process increases the time required to obtain test results and, consequently, to make clinical decisions. In other words, the longer the time required to perform the laboratory test, the longer the time to clinical decision-making and, consequently, the greater the likelihood of the patient developing severe clinical conditions [1].

**Fig. 1:**
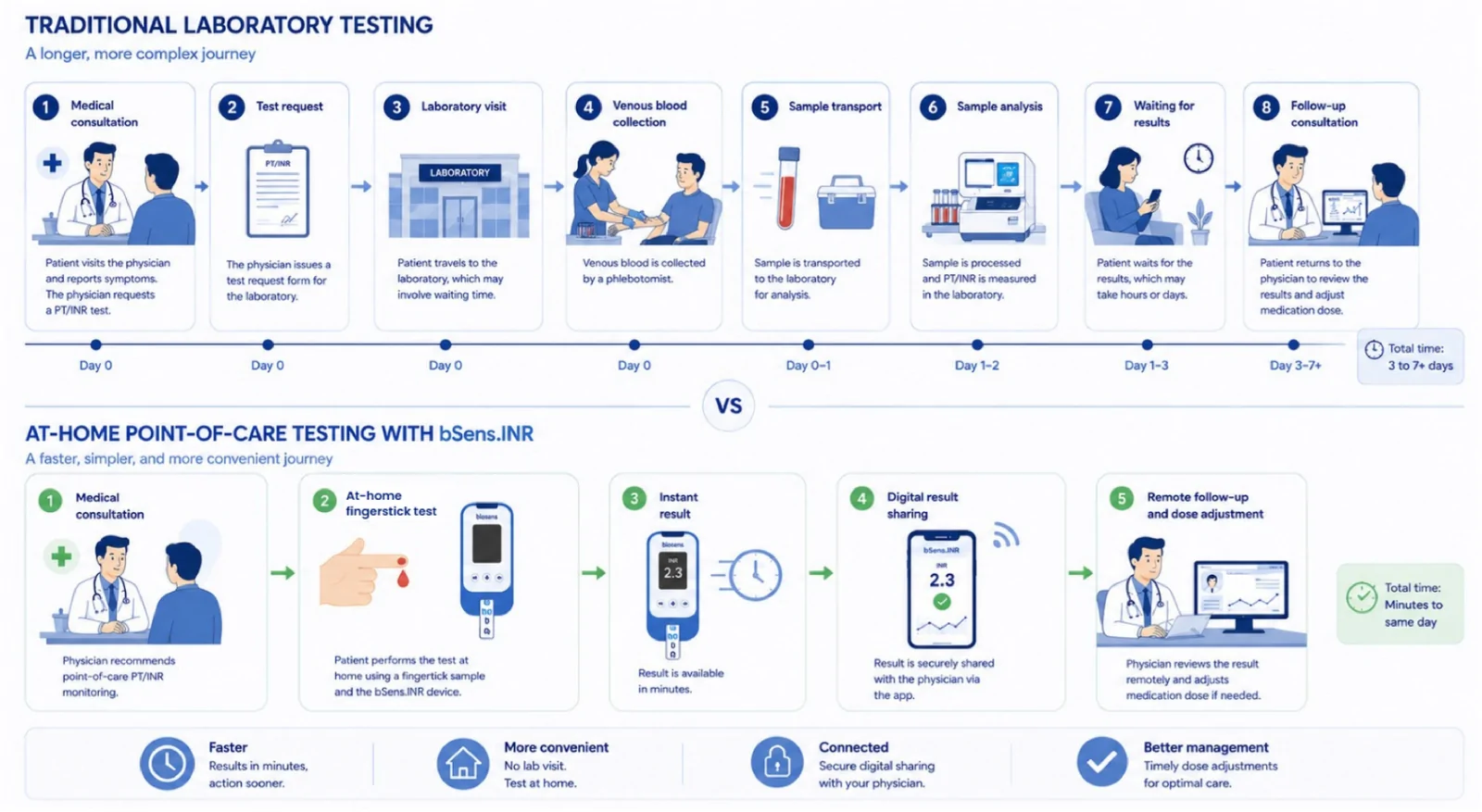
Comparison of the anticoagulated patient journey using the reference laboratory test and point-of-care testing.

Also driven by this challenge, the demand for faster, portable, and easy-to-use diagnostic tests has been increasing each year and represents a global need, although the drivers for adopting these technologies vary depending on the surrounding economic conditions. In high-income countries, the goal is to expedite the diagnostic process, reduce costs, and make healthcare processes more efficient and faster. However, in resource-limited settings, such as low-income countries, remote laboratory tests, also known as Point-of-Care (POC) tests, may be the only viable option when the nearest laboratory is hours away. This scenario represents a significant portion of Brazilian territory, particularly rural areas, where healthcare services and providers lack the infrastructure required to diagnose patients. Therefore, investing in the infrastructure necessary for the industrialization of this technology is essential to enable the introduction of more affordable products into the market. This will facilitate the widespread adoption of these diagnostic tools, improving the quality of healthcare. Such investment can translate into tangible benefits through the reduction of negative outcomes, including morbidity and mortality, length of hospital stay, work absenteeism, and reduced quality of life, while also ensuring economic returns to society [2].

The global market for POC devices for measuring prothrombin time (PT) and the International Normalized Ratio (INR) is projected to grow significantly, with an estimated compound annual growth rate (CAGR) of 8% over the next five years. This topic is a high priority for the World Health Organization (WHO), driven by the increasing prevalence of chronic conditions, such as cardiovascular diseases, which account for approximately one-third of all deaths worldwide, corresponding to approximately 20 million deaths annually [3].

Coagulation disorders associated with these diseases can lead to stroke, pulmonary embolism, myocardial infarction, bleeding, and surgical complications, situations in which rapid decision-making can be life-saving. Therefore, obtaining rapid PT and INR results is essential [1], [4]. In addition, this test is essential for monitoring the safety and efficacy of treatment with vitamin K antagonists, such as warfarin, ensuring that INR values remain within the therapeutic target established by the physician and minimizing the risk of thrombotic and bleeding events [5]. Warfarin therapy remains widely used for the prevention and treatment of thromboembolic events. However, due to its narrow therapeutic window and high interindividual variability in response, inadequate treatment can increase the risk of thromboembolic or hemorrhagic events, significantly affecting patient morbidity and mortality. In this context, periodic INR monitoring is essential to ensure therapeutic safety and efficacy [6], [7]. In self-monitoring, the patient performs the test and communicates the result to the healthcare professional, who determines the appropriate clinical management, as illustrated in Fig. 1, in comparison with the traditional laboratory pathway of an anticoagulated patient. In self-management, after performing the test, the patient adjusts or maintains the medication dose according to predefined values established in consultation with the healthcare professional [6], [7].

In recent years, direct oral anticoagulants (DOACs), including apixaban, rivaroxaban, dabigatran, and edoxaban, have increasingly replaced warfarin for several clinical indications due to their more predictable anticoagulant effects, fewer dietary interactions, and lack of requirement for routine INR monitoring. However, warfarin remains essential for specific patient populations, particularly those with mechanical heart valves. Clinical trials evaluating dabigatran and apixaban in these patients were prematurely terminated due to increased thromboembolic complications compared with warfarin [8], [9]. Therefore, despite the growing use of DOACs, reliable and accessible INR monitoring remains important for patients requiring long-term vitamin K antagonist therapy.

Most individuals are able to adjust their warfarin dose effectively and safely when provided with appropriate instruction and training and when the test demonstrates consistent quality control [10]. The use of POC devices for self-monitoring and self-management of warfarin therapy is recommended by the 2018 guideline on the management of venous thromboembolism issued by the American Society of Hematology, with self-management considered superior to other management strategies. Multiple studies have demonstrated that self-monitoring and self-management of warfarin therapy achieve outcomes that are as good as or better than standard management [11].

The system was developed in accordance with the ASSURED criteria established by the WHO. ASSURED stands for Affordable, Sensitive, Specific, User-friendly, Rapid and Robust, Equipment-free, and Deliverable to end-users. The system is also aligned with the growing demand for rapid, portable, and easy-to-use remote laboratory testing, driven by a combination of factors, including continuous technological advances that enable the miniaturization and portability of devices, fundamental changes in healthcare practices, and the search for more accessible and convenient solutions for health diagnosis and monitoring. The bSens.INR device (Biosens, Brazil) was designed for the assessment of coagulation disorders and monitoring of anticoagulant therapy at the POC [12].

In addition to bSens.INR, which is currently undergoing submission for regulatory approval with the Anvisa, there are several point-of-care systems intended for PT/INR determination available on the national and international markets, including MISSION PT/INR, CoaguChek XS, Cascade POC Analyzer, Coag-Sense, i-STAT PT/INR, Electrometer Plus PT/INR Device, microINR, Portable Coagulation Analyzer, qLabs® PT/INR Q-3, Hemo POC, and LansionBio systems. Published clinical evaluation and validation studies are available for several of these devices, allowing comparison of the methodological strategies employed. In general, clinical performance studies of POC systems for PT/INR monitoring use the International Organization for Standardization (ISO) 17593 - Clinical laboratory testing and in vitro medical devices - Requirements for in vitro monitoring systems for self-testing of oral anticoagulant therapy, as one of the main methodological references. This standard establishes specific requirements for oral anticoagulant therapy monitoring systems, providing criteria related to study design, the number and distribution of samples across different INR ranges, required performance studies, and result acceptability criteria [13], [14], [15], [16].

In general terms, the clinical evaluation involves performing the test on the POC device using capillary whole blood obtained by finger puncture, concomitantly with the collection of venous blood in a sodium citrate tube for PT/INR determination by the reference laboratory method. The results obtained by the two methods are then compared considering the applicable performance and acceptability criteria established by ISO 17593. In addition to assessing compliance with the acceptability criteria, published studies may employ complementary statistical analyses for comparison between methods, including Bland-Altman agreement analysis, association analysis using Pearson correlation, linear regression/relationship between the POC device results and the reference method, and, when applicable, Analysis of Variance (ANOVA) to investigate possible effects of experimental factors, such as different lots, instruments, operators, or days of analysis. These statistical tools complement the performance evaluation, allowing the characterization of agreement, bias, the relationship between methods, and potential sources of system variability [13].

Given the above, this study aims to present and evaluate the data obtained in the validation of the bSens.INR System, conducted based on the recommendations of ISO 17593:2022, as well as to compare the results obtained with published clinical validation studies for POC devices intended for the same purpose of PT/INR monitoring [13], [14].

## 2 Materials and methods

### 2.1 Study design

A prospective diagnostic accuracy study was conducted from March to September 2026 in southern Brazil, with the aim of evaluating the analytical and clinical performance of the bSens.INR System for PT and INR determination (patent application in progress, BR10202600918), by comparing the results obtained with the device with those obtained using a reference laboratory method.

The protocol entitled “Analytical Validation of a Remote Laboratory Test for the Detection of Prothrombin Time Using the bSens.INR System in the Context of the Brazilian Unified Health System (SUS)” was approved by the Research Ethics Committee under opinion No. 81330424.6.0000.5338. Complementary assessments conducted at the company's facilities and in the home setting were carried out within the scope of a project approved under opinion No. 83945318.0.0000.5344, ensuring that all activities were conducted in accordance with the applicable ethical requirements for research involving human participants.

The validation was conducted in accordance with the criteria and procedures established in ISO 17593:2022, which is applicable to prothrombin time monitoring systems and INR determination [13]. The evaluation encompassed different aspects of bSens.INR performance, including repeatability, intermediate precision, accuracy, and usability, as well as a comparison of the results obtained with the device and those obtained using the reference laboratory method. Overall, the study design was structured into sequential stages, as presented in Fig. 2.

**Fig. 2:**
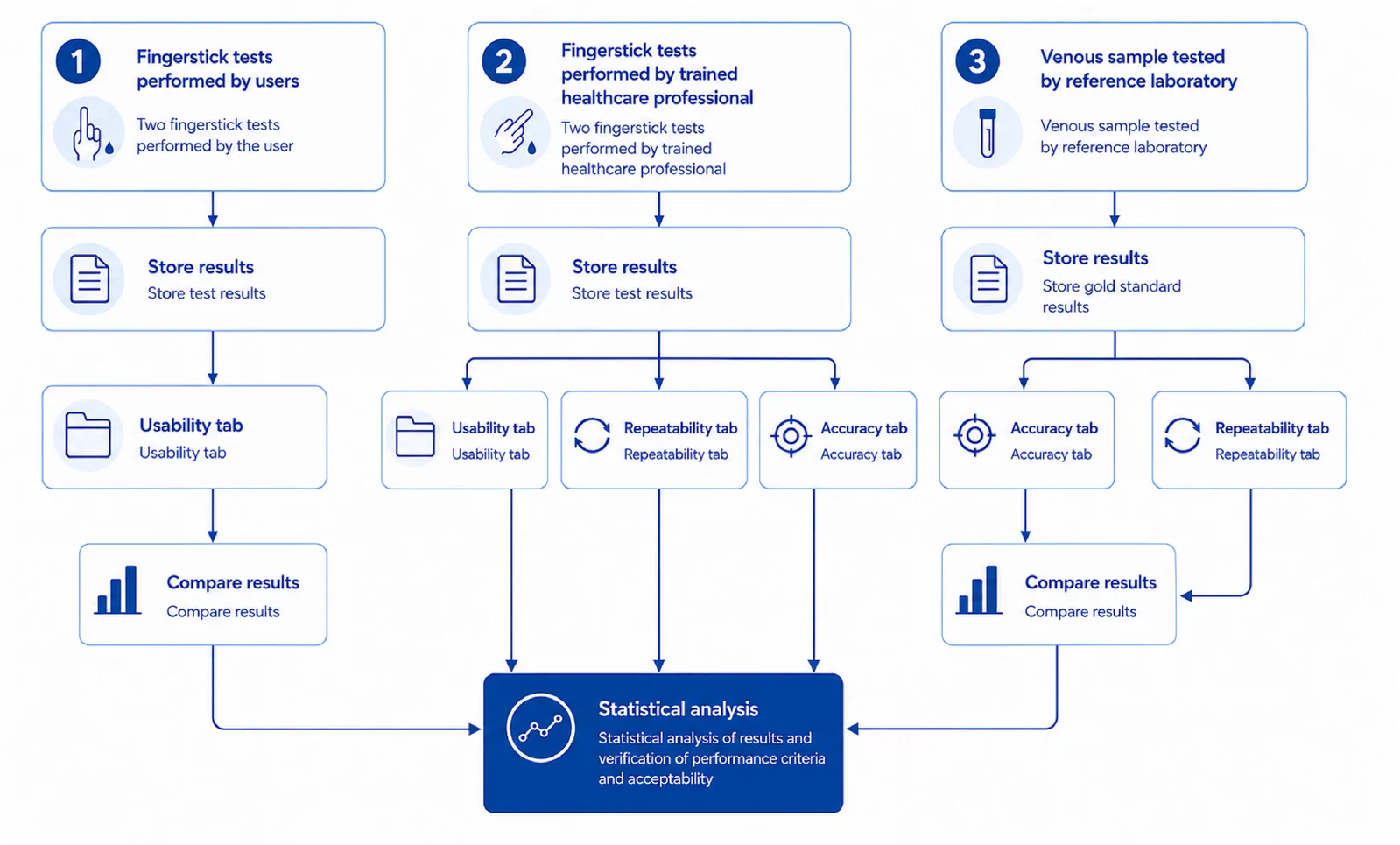
Representative flowchart of the experimental and analytical steps performed throughout the study.

### 2.2 Study population

The study population consisted of adults (≥18 years) who, after being invited by the validation team and adequately informed about the study objectives and procedures, voluntarily agreed to participate and signed the Informed Consent Form (ICF). Participants receiving oral anticoagulant therapy with warfarin, encompassing different levels of anticoagulation, as well as individuals not receiving warfarin therapy, were included. This population composition was established to encompass different INR values within the measurement range of interest of the device and to allow evaluation of its performance across this range.

Participants who did not meet the eligibility criteria or who presented conditions that prevented the adequate collection of the samples required for testing were excluded from the study. These conditions included difficulty in obtaining venous access and insufficient blood volume, both for analysis of the venous sample and for performing the test using capillary blood obtained by fingerstick, which could compromise the proper execution of the procedures or the comparison between methods.

Additionally, results that did not meet the criteria previously established in the validation protocol were excluded from the analysis. For participants receiving warfarin therapy, an INR value of ≥2.0 was established as an inclusion criterion for analysis, with a variation of up to 20% from this threshold considered acceptable, as previously defined in the protocol.

The confidentiality and privacy of all participants included in the study were preserved. To ensure the anonymization of the reported data, each participant was assigned a sequential identification code according to the order of enrollment, starting with P001.

### 2.3 Demographic and clinical characterization

To characterize the study population, a questionnaire was administered prior to sample collection to obtain demographic and clinical information, including age, sex, race/ethnicity, presence of relevant chronic diseases, medications, and duration of warfarin use, when applicable.

### 2.4 bSens.INR system

The bSens.INR System is a rapid and portable test classified as a Class III in vitro diagnostic medical device, according to Rule 3(f) of Collegiate Board Resolution (RDC) No. 830/2023. It was developed to be intuitive and accessible, allowing individuals without specialized knowledge or technical expertise to use the system easily. The device provides rapid and accurate quantitative measurements of PT and INR using approximately 8 μL of capillary blood collected by fingerstick. This technological solution is intended both for individuals receiving oral anticoagulant therapy, such as warfarin, and for healthcare professionals who require efficient assessment of coagulation disorders [17].

The bSens.INR System consists of a reactive strip and an electronic reader that clearly guides the user through all stages of the test. As illustrated in Fig. 3, the reactive strip contains an immobilized reagent composed of recombinant human tissue factor, thromboplastin, calcium, buffers, and stabilizers. When the capillary blood sample is applied to the reactive strip and comes into contact with the reagent, the coagulation process is activated, promoting thrombin formation. The electronic reader heats the reactive strip, stimulates the reaction, and detects the coagulation process through a time-dependent electrochemical impedance measurement. The resulting signal is processed by an algorithm that converts the measured electrical signal into clotting time in seconds and INR values. In addition, the device stores the results in its internal memory, facilitating continuous monitoring by both patients and healthcare professionals.

**Fig. 3:**
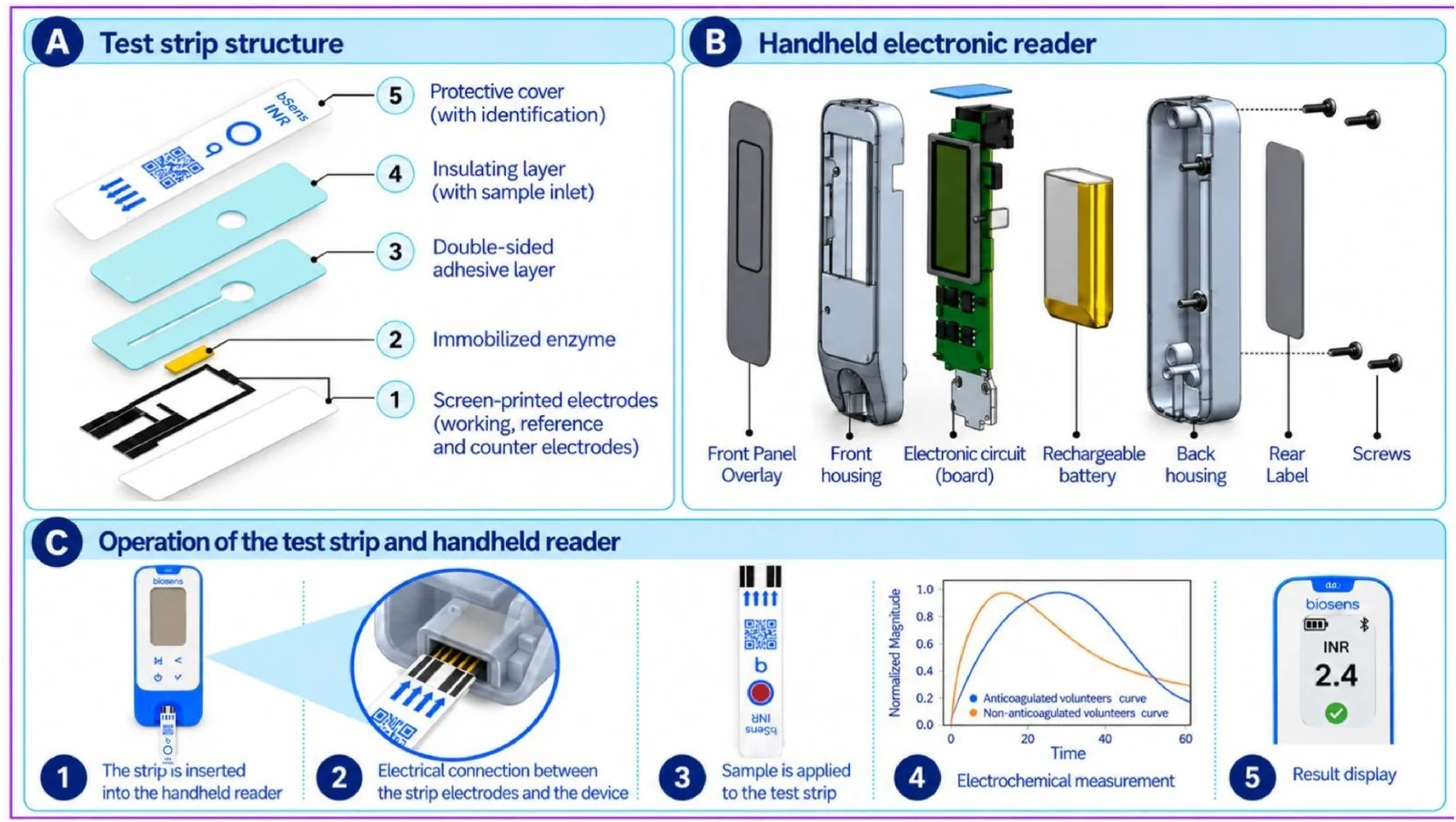
Structural components and operating principle of the bSens.INR.

The bSens.INR system was calibrated considering three main parameters: operating temperature, International Sensitivity Index (ISI), and Mean Normal Prothrombin Time (MNPT). These parameters were configured directly in the electronic device's software. The operating temperature was established through performance tests conducted under different conditions, with 33.5 °C selected because it provided the best agreement with the laboratory reference method (data not shown). The ISI was set to 1.0 based on the information provided in the manufacturer's instructions for the human-derived thromboplastin reagent, while the MNPT was established at 12.9 s based on the mean PT values obtained from individuals not receiving anticoagulant therapy.

For the validation tests of the bSens.INR System, a qualified professional initially collected capillary blood by fingerstick. Immediately after collection, the sample was applied to the reactive strip inserted into the device, following the previously established test procedure. At the end of each measurement, the PT/INR values provided by the bSens.INR were recorded and linked to the corresponding participant identification code. For each participant, measurements with the bSens.INR were performed in duplicate. The sample collection and measurement procedures are illustrated in Fig. 4.

**Fig. 4:**
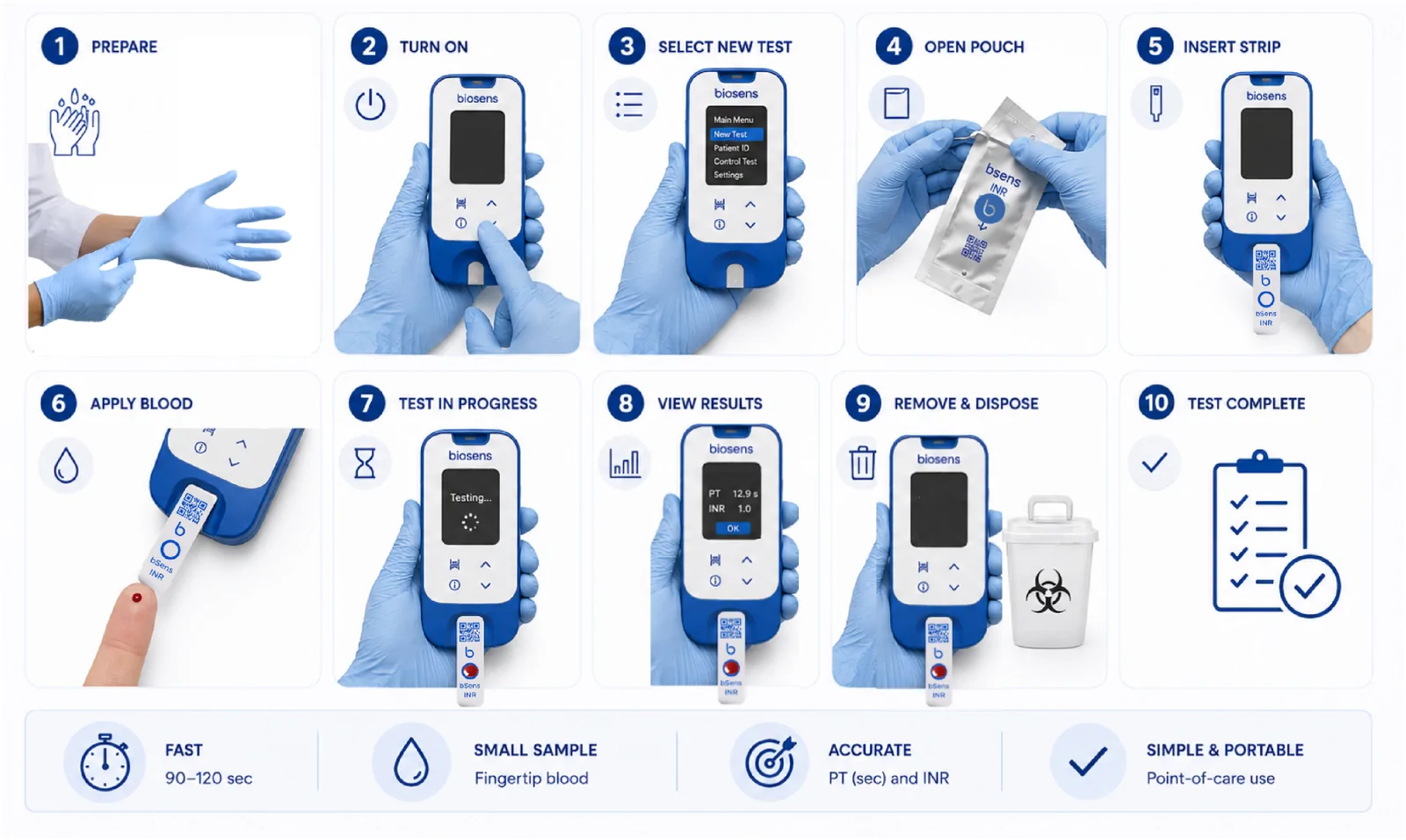
Sequence of steps for testing with the bSens.INR system.

### 2.5 Reference laboratory method

Subsequently, venous blood samples were collected from the participants by venipuncture using tubes containing 3.2% sodium citrate (0.109 mol/L) as the anticoagulant to determine the reference PT/INR values for comparison with the results obtained using the bSens.INR System. Sample collection and processing were performed in accordance with established laboratory procedures to preserve the pre-analytical conditions required for accurate determination of coagulation parameters.

The venous blood samples collected in tubes containing sodium citrate were sent for analysis using the reference laboratory method. Two coagulation systems were used in this study: CoagMaster 2.0 (WAMA Diagnóstica), using the TP Wama kit, and START Max (Diagnostica Stago), using the STA-NeoPTimal reagent. Both reagents contain rabbit brain-derived thromboplastin. For the CoagMaster 2.0 system, the ISI ranged from 1.0 to 1.2, while the MNPT ranged from 13.5 to 14.6 s. For the START Max system, the ISI also ranged from 1.0 to 1.2 during the study period, while the MNPT was 14.0 s. The reference analyses were performed by laboratories participating in the Brazilian National Quality Control Program (Programa Nacional de Controle de Qualidade – PNCQ), an external quality assessment program for clinical laboratories. However, detailed information regarding the laboratories' internal calibration procedures and individual external quality assessment performance reports was not available to the authors.

For PT determination, the citrated venous blood samples were initially centrifuged to obtain plasma. The plasma was subsequently incubated at approximately 37 °C and analyzed using the coagulometer following the addition of a calcium-containing thromboplastin reagent. The addition of calcium allows recalcification of the citrated plasma and, together with thromboplastin, promotes activation of the extrinsic and common coagulation pathways, culminating in clot formation. Clot formation was automatically detected by the coagulation system according to the specific detection principle of each instrument, based on changes in the physical properties of the sample during fibrin formation, monitored through the movement of a metallic element and a magnetic detection system.

The results obtained using the reference laboratory method were recorded in laboratory reports and expressed as PT/INR values, which were subsequently used for comparison with the results obtained using the bSens.INR System. For each determination performed with the bSens.INR, the corresponding result obtained using the reference laboratory method was established, considering the same sample or a collection performed at the same time, in accordance with the experimental procedure defined in the protocol.

The results were paired individually, allowing direct comparison between the INR value obtained using the bSens.INR and the corresponding value determined by the reference laboratory method. Each pair of results was identified and recorded for subsequent statistical analysis of agreement and accuracy, including assessment according to the acceptance criteria established by ISO 17593:2022 [13].

### 2.6 Clinical performance studies

#### 2.6.1 Accuracy

The accuracy assay aimed to evaluate the performance of the bSens.INR System by assessing the agreement between the INR results obtained with the device and those determined using the reference laboratory method. A total of 200 samples were included in this assay, comprising 170 samples from volunteers receiving warfarin therapy and 30 samples from volunteers who were not receiving vitamin K antagonist anticoagulant therapy. In this assay, a participant receiving anticoagulant therapy could provide samples on more than one occasion throughout the study, provided that the collections were performed on different days. This approach was adopted considering that the response to anticoagulant therapy may vary over time due to factors such as warfarin dose adjustments, diet, concomitant medication use, clinical status, and lifestyle habits, which may influence PT and INR values and, consequently, the patient's therapeutic range. In cases where the same participant was evaluated on more than one occasion, the original identification code was retained, preventing the participant from being counted as a new individual and allowing the different assessments associated with the same participant to be identified. Each test was considered a single determination for the corresponding performance analyses, while the number of individuals was counted separately for population characterization. This approach allowed the number of observations available for assessing agreement between the bSens.INR and the reference method to be increased without considering each repeated determination as an independent participant.

Accordingly, capillary blood samples were collected in duplicate and tested using the bSens.INR System. For comparison with the reference method, a venous blood sample was collected from each participant and analyzed using the reference method described above to assess accuracy through comparison between the two methods. The distribution of samples across INR ranges was performed according to Table 1, following the recommendations of ISO 17593:2022 [13].

**Table 1:** INR ranges and sample fractions for the accuracy study.

	INR range	Fraction of Samples Recommended by ISO	Number of Samples
Samples from volunteers not receiving anticoagulant therapy	<2.0	10-15%	30
Samples from volunteers receiving warfarin therapy	2.0 a 2.8	15-40%	80
	>2.8 a 3.7	15-40%	70
	>3.7 a 4.5	10-30%	20

For data analysis, method accuracy was considered satisfactory when at least 95% of the results obtained from capillary blood samples were within the acceptance limits specified by ISO 17593:2022. The acceptance limits for bSens.INR results compared with the reference method were ±0.4 INR units for INR values < 2.0 and ± 20% for INR values ranging from 2.0 to 4.5 [13].

In addition, the correlation between the INR results obtained using the reference method and those obtained with the bSens.INR System was evaluated using Pearson correlation analysis and linear regression, while agreement between the methods was assessed using the Bland-Altman method. The Bland-Altman analysis calculates the mean difference between two measurement methods and the 95% limits of agreement, defined as the mean difference ± 2 standard deviations (SD). The Bland-Altman plot consists of two axes: the horizontal (x) axis represents the mean of the two measurements obtained by the compared methods, calculated as (Measurement 1 + Measurement 2)/2, while the vertical (y) axis represents the difference between the paired measurements, calculated as Measurement 1 - Measurement 2 [18], [19].

#### 2.6.2 Precision (repeatability)

The repeatability assay aimed to evaluate the precision of the bSens.INR System by assessing the agreement between INR results obtained from repeated measurements of the same sample under the same test conditions. A total of 60 volunteers were included in this assay, comprising 45 volunteers receiving warfarin therapy and 15 volunteers who were not receiving vitamin K antagonist anticoagulant therapy. The requirements specified in ISO 17593:2022 were also applied to these assays [13].

Accordingly, capillary blood samples were collected in duplicate and tested using the bSens.INR System. For comparison with the reference method, a venous blood sample was collected from each participant and analyzed using the reference method described above to assess accuracy through comparison between the two methods. The distribution of samples from the volunteers across the different INR ranges was performed according to Table 2, following the recommendations of ISO 17593:2022.

**Table 2:** INR ranges for the repeatability study.

	Interval	INR range	Minimum Number of Patients
Volunteers not receiving anticoagulant therapy	Non-therapeutic	<2.0	15
Volunteers receiving anticoagulant therapy	Low therapeutic	2.0 a 3.0	25
	High therapeutic	>3.0 a 4.5	20

For data analysis, the difference between the duplicate measurements and the acceptable range were calculated according to Equation (1), where 0.10 represents the acceptable 10% variation between duplicate measurements and 4.2 is a constant in the equation.

Equation 1 Acceptablecalculatedinterval=meanoftheduplicatemeasurementsx0.10x4.2

The result was considered repeatable when the actual difference between the duplicate measurements was lower than the calculated acceptable interval, as specified in ISO 17593:2022 [13].

#### 2.6.3 Intermediate precision

The intermediate precision assay aimed to evaluate the ability of the bSens.INR System to provide consistent INR results under variations in test conditions, such as different days, strip lots, and devices. For this assessment, three volunteers with INR values below 2.0 and not receiving anticoagulant therapy were included. As the bSens.INR System does not have commercially available control material, the use of samples from these volunteers allowed different devices and reactive strip lots to be evaluated using blood samples with similar INR values.

The assays were performed over five days using three electronic devices, designated Device 1, Device 2, and Device 3, and three lots of reactive strips, designated Lot 1, Lot 2, and Lot 3, following the recommendations of ISO 17593:2022. For each lot of reactive strips, two determinations were performed on each of the three devices, resulting in six determinations per lot per day. Thus, 18 determinations were performed per day, totaling 90 determinations over the five-day period. Fig. 5 presents the experimental design used in the study.

**Fig. 5:**
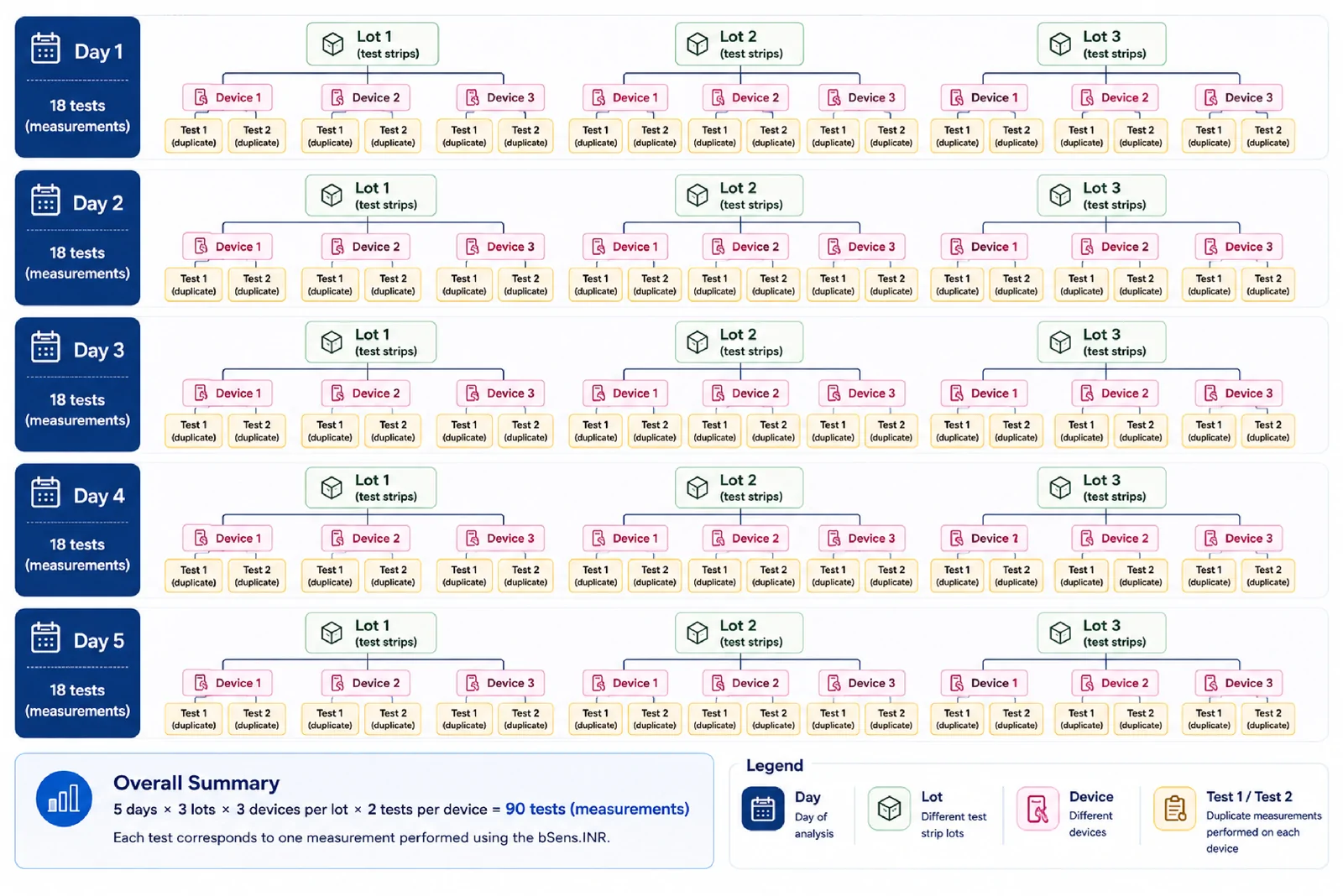
Example diagram of the intermediate precision study.

For data analysis, each combination of testing day and reactive strip lot was considered a block, resulting in six determinations per block. For each block, the six results obtained were used to calculate the mean, standard deviation, and upper (UL) and lower (LL) acceptance limits, according to Equations Equation 2, Equation 3).

Equation 2 UL=(Maximumvalue−Mean)/Standarddeviation

Equation 3 LL=(Mean−Minimumvalue)/Standarddeviation

Following the recommendations of ISO 17593:2022, when any of the values obtained from the calculations exceeds 2.48, the corresponding test condition is considered to have failed. In addition, to assess the influence of different sources of variation on the agreement between the results obtained with the bSens.INR and those obtained using the reference laboratory method, the deviation between the bSens.INR result and the corresponding result obtained using the reference method was calculated for each determination. The calculated deviations were analyzed considering the following sources of variation: instrument used, test day, reactive strip lot, and replicate. For each factor, ANOVA was performed to determine whether the different levels of each factor were associated with statistically significant differences in the deviations observed relative to the reference method [13], [20].

The analyses were performed using a significance level of 5% (α = 0.05). As an acceptance criterion, a statistically significant influence of the evaluated factor was considered absent when the p-value was greater than 0.05. Under this condition, there was no statistical evidence that the respective factor systematically contributed to the variation in the deviation between the results obtained with the bSens.INR and those obtained using the reference laboratory method.

#### 2.6.4 Usability and self-monitoring

The usability test aimed to evaluate whether the intended user was able to correctly operate the bSens.INR System and obtain INR results in agreement with those obtained by a qualified professional, assessing whether test performance by the user introduced errors that could compromise result reliability. A total of 80 samples from 40 volunteers with INR values within the target range of 2.0 to 4.5 were included in this assay, following the recommendations of ISO 17593:2022. Some volunteers participated in the assessment on more than one occasion, contributing more than one evaluation throughout the study. Initially, each volunteer received prior instructions from a healthcare professional on the use of the bSens.INR System and test performance. The professional then demonstrated the procedure by performing two tests on the volunteer. Following this step, the device was made available to the volunteer, who independently performed two self-tests according to the instructions provided [13].

For data analysis, the mean, variance, standard deviation, and coefficient of variation (CV) were calculated for the duplicate measurements performed by the healthcare professional and the lay user. As an acceptance criterion, the CV of the results obtained from tests performed by lay users and healthcare professionals was required to be below 10%, in accordance with the acceptance criterion also established for the Precision Assay (repeatability).

### 2.7 Evaluation of potential interferents

The assessment of potential interfering factors aimed to identify and describe clinical, hematological, and pre-analytical characteristics present in the study population that could be associated with changes in the performance of the bSens.INR System. An exploratory and descriptive analysis of potential factors that could interfere with the performance of the bSens.INR System was conducted. Clinical, hematological, medication-related, and sample quality information available for the study participants was considered.

When available, hematocrit values, platelet counts, and hemoglobin concentrations were evaluated, as well as the presence of chronic diseases, antiphospholipid syndrome (APS), and concomitant medication use, with particular attention to furosemide use. Pre-analytical characteristics observed in the venous samples were also recorded, including the presence of hemolysis and lipemia.

To further investigate the potential influence of hematocrit on the analytical performance of the bSens.INR System, hematocrit values were correlated with the differences between bSens.INR measurements and the laboratory reference method. For each determination, measurement bias was calculated as the difference between the mean of two bSens.INR replicate measurements and the corresponding laboratory INR result. Spearman's rank correlation coefficient (ρ) was used to assess the association between hematocrit and measurement bias. As some participants contributed multiple measurements, an additional analysis was performed using the mean measurement bias calculated for each participant, thereby reducing the influence of repeated observations from the same individual. Statistical significance was established at p < 0.05. Additionally, a Bland–Altman plot was used to visually assess measurement agreement between the bSens.INR System and the laboratory reference method, highlighting measurements from participants with low hematocrit values.

The remaining potential interfering factors were assessed using descriptive and frequency analyses, considering their occurrence in the study population and, on an exploratory basis, their presence among cases in which greater differences were observed between the results obtained with the bSens.INR System and those obtained using the reference laboratory method. Specific inferential statistical tests were not performed for these additional factors due to the limited number of observations involving certain clinical conditions or potential interferents. Therefore, these findings were used to identify potential factors requiring attention and to support further investigations, rather than being interpreted as conclusive evidence of interference with INR determination.

## 3 Results

### 3.1 Characterization of the population

A total of 77 participants were included in the study, of whom 30 were not receiving anticoagulant therapy and 47 were receiving such therapy. Among the participants receiving anticoagulant therapy, some underwent more than one sample collection throughout the study. However, for population characterization, each participant was counted only once, regardless of the number of collections performed. The characteristics of the study population, including the distribution of chronic diseases, medications used, and duration of anticoagulant therapy, are presented in Table 3.

**Table 3:** Characterization of the study population.

Variable	Category	n (%)	Category	n (%)
Sex	Female	39 (50.6%)	Male	38 (49.4%)

Race	White	71 (92.2%)	Mixed-race	4 (5.2%)
	Black	2 (2.6%)		
Age Group	20-29 years	13 (16.9%)	50-59 years	13 (16.9%)
	30-39 years	12 (15.6%)	60-69 years	19 (24.7%)
	40-49 years	13 (16.9%)	≥70 years	7 (9.1%)
Diseases	Hypertension	13 (33.3%)	Marfan syndrome	1 (2.6%)
	Diabetes	8 (20.5%)	Pulmonary hypertension	1 (2.6%)
	Dyslipidemia	7 (17.9%)	Thyroid disorder	1 (2.6%)
	APS	5 (12.8%)	Heart failure	1 (2.6%)
	Prediabetes	4 (10.3%)	Hypothyroidism	1 (2.6%)
	Arrhythmia	3 (7.7%)	HIV	1 (2.6%)
	Fibromyalgia	2 (5.1%)	Atrial fibrillation	1 (2.6%)
	Gout	2 (5.1%)	Factor V deficiency	1 (2.6%)
	Lupus	2 (5.1%)	Rheumatic disease	1 (2.6%)
	Epilepsia	1 (2.6%)	Thromboangiitis obliterans	1 (2.6%)
			Thrombophilia	1 (2.6%)
Medications	Metoprolol	19 (24.7%)	Fluoxetine	3 (3.9%)
	Simvastatin	13 (16.9%)	Spironolactone	3 (3.9%)
	Enalapril	11 (14.3%)	Levothyroxine	3 (3.9%)
	Losartan	9 (11.7%)	Escitalopram	2 (2.6%)
	Hydrochlorothiazide	9 (11.7%)	Valsartan	2 (2.6%)
	Furosemide	7 (9.1%)	Amiodarone	2 (2.6%)
	Amlodipine	6 (7.8%)	Insulin	2 (2.6%)
	Metformin	6 (7.8%)	Verapamil	2 (2.6%)
	Omeprazole	5 (6.5%)	Cilostazol	2 (2.6%)
	Acetylsalicylic acid	4 (5.2%)	Hydroxychloroquine	2 (2.6%)
	Carbamazepine	4 (5.2%)	Calcium	2 (2.6%)
	Rosuvastatin	4 (5.2%)	Doxazosin	2 (2.6%)
	Oral contraceptive	3 (3.9%)	Others	1 (1.3%)
Warfarin	No	30 (39%)	Yes	47 (61%)

Duration of Use	Less than 1 year	4 (8.5%)	11-20 years	12 (25.5%)
	1-5 years	10 (21.3%)	More than 20 years	2 (4.3%)
	6-10 years	19 (40.4%)		

Among the 77 participants included in the study, 39 had chronic diseases (50.6%). Of these, 4 (5.2%) belonged to the group not receiving anticoagulant therapy, while the remaining participants belonged to the warfarin therapy group. Among the 38 participants without chronic diseases (49.4%), 12 (15.6%) belonged to the anticoagulant therapy group. These data indicate a higher frequency of chronic diseases among participants receiving anticoagulant therapy.

A total of 66 distinct medications or therapeutic descriptions were identified, with a higher frequency of use among participants receiving anticoagulant therapy. Among the 47 participants in this group, only 4 were not taking any medication. In the group of 30 participants who were not receiving warfarin, 21 were not taking any medications. Thus, concomitant medication use was more frequent among participants receiving anticoagulant therapy.

The least frequently used medications were topiramate, dutasteride, prednisolone, the combination of dutasteride and tamsulosin hydrochloride, abiraterone, pregabalin, lamotrigine, pantoprazole, nortriptyline, riociguat, formoterol fumarate and budesonide, ambrisentan, carvedilol, imipramine, phenobarbital, diazepam, itraconazole, isosorbide, digoxin, ursodeoxycholic acid, nitrofurantoin, tirzepatide, bisoprolol, antiretroviral medication, paracetamol, trazodone, bromazepam, sumatriptan, atenolol, amitriptyline, propranolol, nebivolol, golimumab, vitamin A, risperidone, dapagliflozin, atorvastatin, ezetimibe, vitamin B12, leflunomide, and duloxetine. Each of these medications was used by one participant, corresponding to 1.3% of the study population. It should be noted that some participants were unable to provide detailed information about all medications they were taking.

Regarding treatment duration, among the 47 participants receiving anticoagulant therapy, the highest frequency was observed among individuals with 6 to 20 years of treatment, corresponding to the 6-10-year and 11-20-year categories.

### 3.2 bSens.INR accuracy

Of the 200 tests performed in duplicate, 197 met the accuracy acceptance criteria (98.5%), while 3 were rejected (1.5%) based on the acceptable range calculated from the reference method results. In addition, among the 200 tests performed in duplicate, 199 met the repeatability criteria (99.5%), and 1 were rejected (0.5%). The mean bSens.INR result showed an overall bias of −0.178 INR relative to the reference method, indicating a general tendency of the bSens.INR to produce slightly lower results than the laboratory method. Among the three tests rejected for accuracy, all three yielded results lower than those obtained using the reference method. All three underestimation errors occurred in the higher INR ranges, particularly above 2.8. Therefore, when the bSens.INR showed an error large enough to result in rejection, underestimation of INR predominated, especially at higher INR values. The **>3.7–4.5** range had the highest proportion of accuracy errors, and a progressive increase in negative bias was also observed as INR increased. Table 4 presents a detailed analysis of accuracy, rejection, and the differences between the reference method and the bSens.INR across the different INR ranges.

**Table 4:** Accuracy and difference between the gold-standard method and bSens.INR.

INR Ranges	Tests	Rejections	Accuracy (%)	Higher Errors	Lower Errors	Mean INR Difference Relative to the Reference Method
<2.0	30	0	100	0	0	−0.040
≥2.0 a 2.8	80	0	100	0	0	−0.035
>2.8 a 3.7	70	1	98.6	0	1	−0.304
>3.7 a 4.5	20	2	90	0	2	−0.513

The correlation between the results obtained with the bSens.INR and those obtained using the reference method was evaluated using the 400 determinations performed, corresponding to duplicate measurements of the 200 samples included in the study. Pearson correlation analysis yielded a correlation coefficient of r = 0.927, indicating a very strong positive linear correlation between the bSens.INR and the reference method, based on the correlation strength interpretation table.

Linear regression, with the reference method values on the X-axis and the bSens.INR results on the Y-axis, was described by Equation (4).

Equation 4 $${INR}_{bSens.INR}=0.8143x{INR}_{Padrão-ouro}+0.3072$$

A slope coefficient below 1 indicates that, as the INR values obtained using the reference method increase, the bSens.INR tends to produce proportionally lower results, indicating progressive underestimation at higher INR values. Graph 1 presents the linear correlation between the results obtained by the two methods, with a slope of ≅ 0.814 and intercept ≅ 0.307.

**Graph 1:**
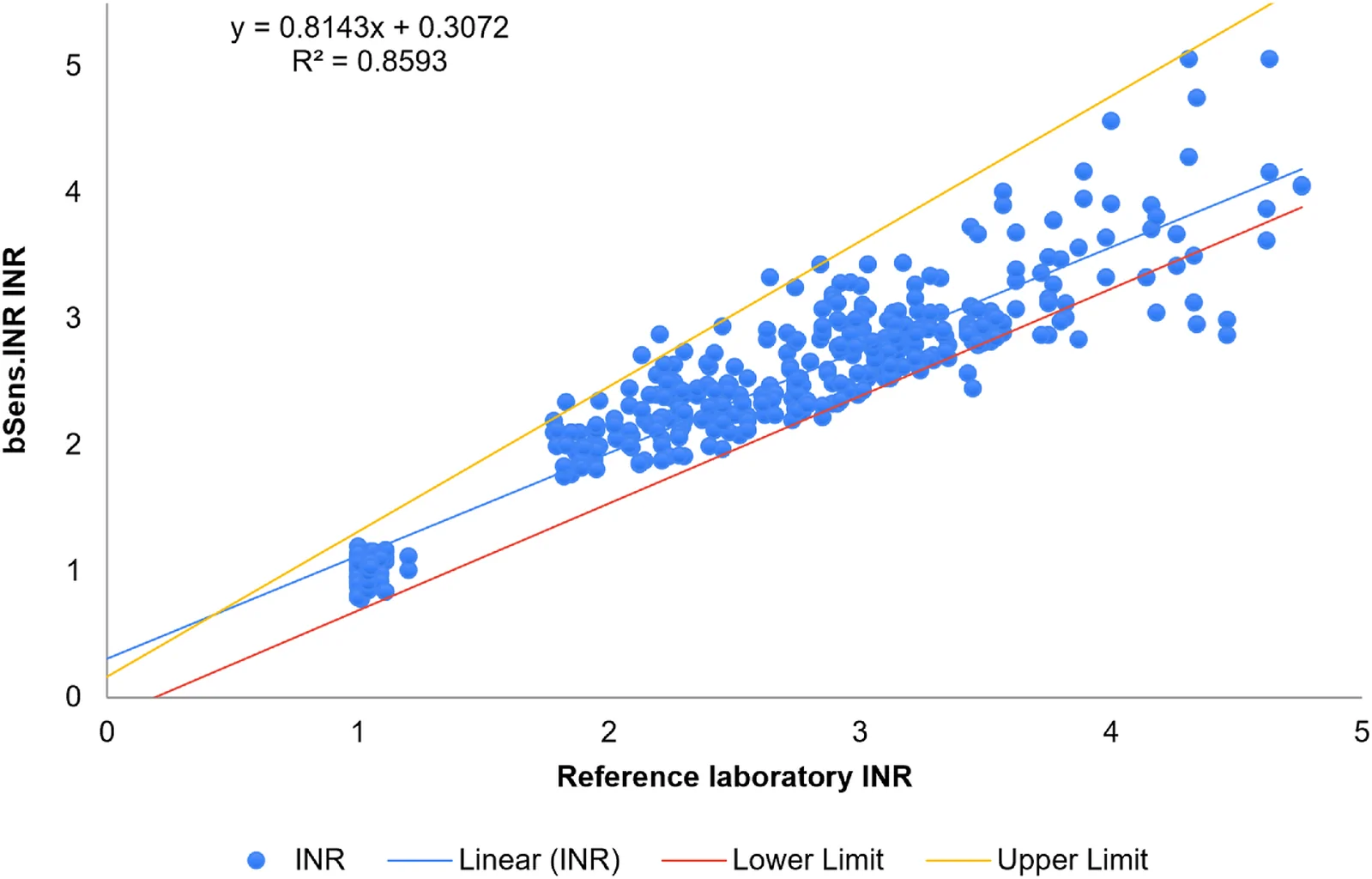
Linear correlation between the methods.

Graph 2 illustrates the difference between the compared methods as a function of INR, considering the overall bias. In this global analysis, 4.5% of the results remained outside the limits of agreement, indicating that 95.5% of the measurements were within the established limits. This demonstrates good agreement between the bSens.INR and the reference method across the entire measurement range. Graph 3 presents the Bland-Altman analysis across the entire measurement range, with the corresponding limits of agreement defined for each INR interval based on the calculated bias.

**Graph 2:**
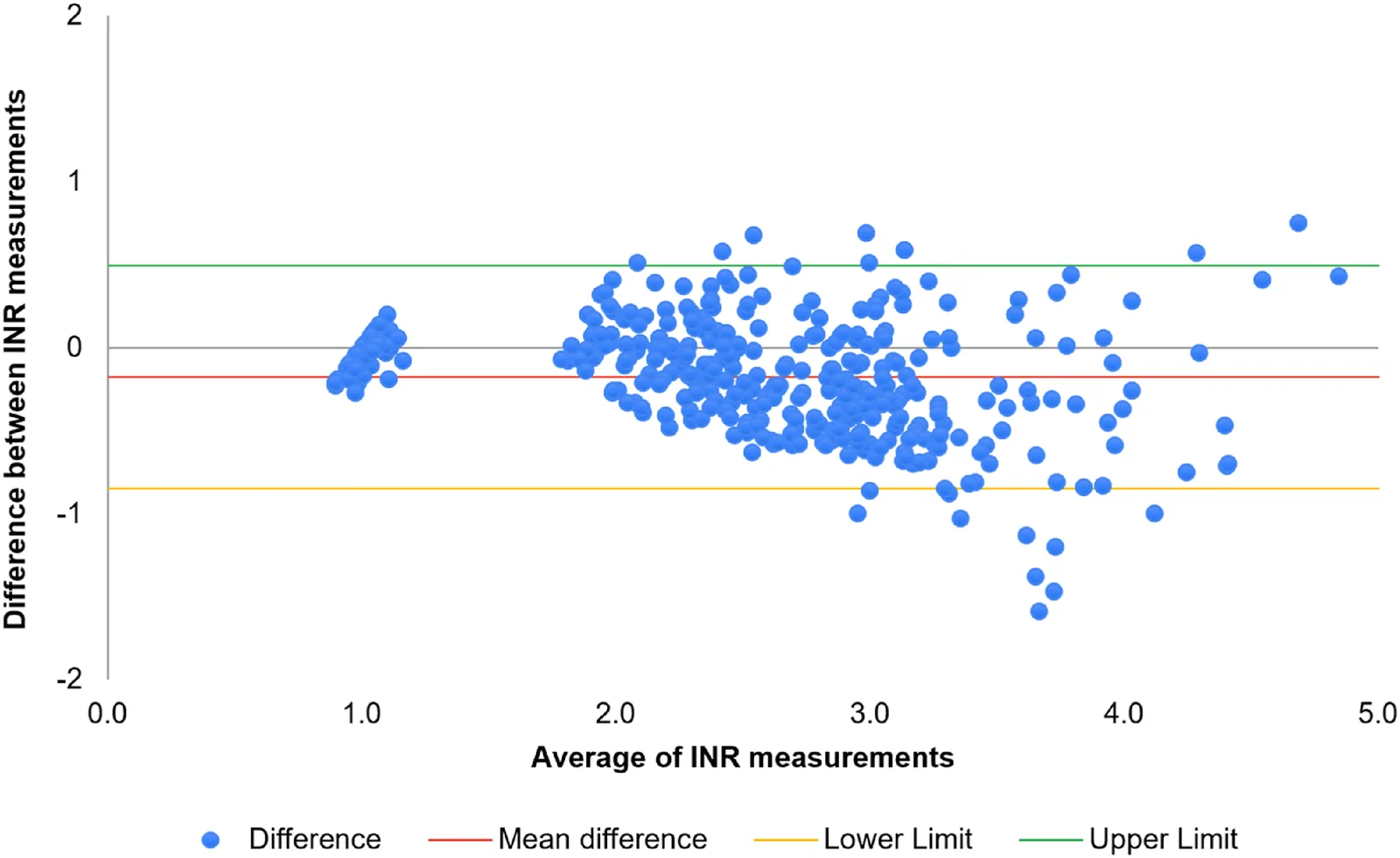
Bland-Altman analysis of the difference between the compared methods as a function of INR values, considering the overall bias.

**Graph 3:**
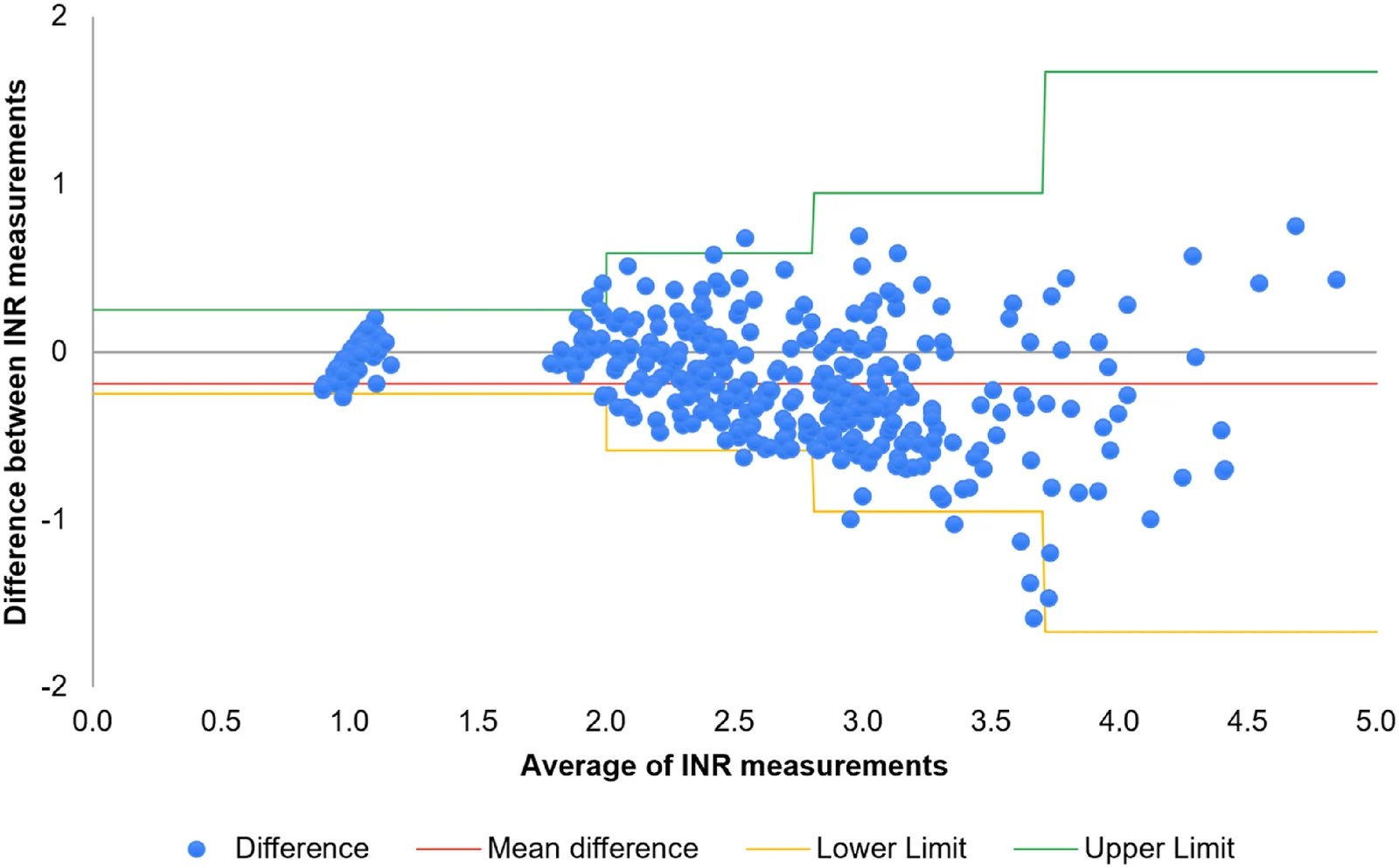
Bland-Altman analysis of the difference between the compared methods as a function of INR values, considering the bias by INR range.

The results obtained demonstrate that the bSens.INR showed satisfactory accuracy performance compared with the reference laboratory method, considering the established measurement range of up to an INR of 4.5.

### 3.3 Precision/repeatability

Of the 60 tests performed in duplicate, corresponding to 60 volunteers distributed across different INR ranges, 100% met the acceptance criteria established for repeatability, demonstrating the ability of the bSens.INR System to provide consistent results in repeated measurements under the same test conditions. For each INR range evaluated, the mean, variance, standard deviation, and coefficient of variation were calculated, and the results are presented in Table 5.

**Table 5:** Measurement precision study results.

INR Range	Test	Mean	Variance	Standard Deviation	Coefficient of Variation
<2.0	15	1.0	0.006	0.07	7.24%
2.0 a 3.0	25	2.3	0.040	0.20	8.78%
>3.0 a 4.5	20	3.0	0.027	0.16	5.53%

### 3.4 Intermediate precision

For all analyses performed, the mean, standard deviation, and coefficient of variation are presented in Table 6. All tests (100%) met the acceptance criteria for intermediate precision, demonstrating adequate reproducibility of the measurement system when different lots of reactive strips, different electronic reader devices, and different analysis days were used.

**Table 6:** Intermediate precision study results.

Day	Strip Lot	Test	Mean	Standard Deviation	Coefficient of Variation
1	1	6	0.98	0.16	15.9%
2	1	6	0.90	0.05	6.07%
3	1	6	0.94	0.09	10.11%
4	1	6	0.94	0.05	5.51%
5	1	6	0.93	0.10	10.19%
1	2	6	1.05	0.09	8.32%
2	2	6	1.05	0.13	12.45%
3	2	6	1.01	0.09	8.50%
4	2	6	1.02	0.07	7.35%
5	2	6	1.02	0.07	6.74%
1	3	6	0.97	0.13	12.88%
2	3	6	0.97	0.08	7.86%
3	3	6	1.01	0.10	9.53%
4	3	6	0.99	0.07	7.21%
5	3	6	0.99	0.09	9.12%

Analysis of variance did not identify statistically significant differences in the deviation of the bSens.INR results from the reference laboratory method associated with the instrument used (F = 1.168; p = 0.300), test day (F = 0.822; p = 0.734), reactive strip lot (F = 1.152; p = 0.315), or replicate measurement (F = 0.900; p = 0.629). All evaluated factors showed p-values above the 5% significance level, with no statistical evidence that these sources of variation systematically affected the agreement between the bSens.INR results and the reference method.

### 3.5 Usability and self-monitoring

The tests yielded 100% of results within the acceptable limits, demonstrating that the bSens.INR device can be successfully used by lay users who have received prior instructions. The mean variance, standard deviation, mean, and coefficient of variation are presented in Table 7.

**Table 7:** Usability study results.

User	Test	Mean	Variance	Standard Deviation	Coefficient of Variation
Lay Users	80	2.50	0.014	0.12	4.71%
Healthcare Professionals	80	2.50	0.025	0.16	6.27%

### 3.6 Potential interfering factors and analysis of discordant results

In the present study, specificity was not directly evaluated through specific interference studies (e.g., assessment using isolated exogenous and endogenous substances). However, accuracy studies were conducted by comparison with a duly validated reference method, without prior exclusion of potential interfering factors related to medications, the biological matrix, or the clinical conditions of the participants. These analyses demonstrated adequate agreement between the results obtained.

The study population showed substantial clinical heterogeneity, including individuals with different pathological conditions and comorbidities. The conditions identified included systemic arterial hypertension, diabetes mellitus, dyslipidemia, APS, prediabetes, arrhythmias, lupus, fibromyalgia, gout, epilepsy, Marfan syndrome, pulmonary hypertension, heart failure, hypothyroidism, human immunodeficiency virus (HIV) infection, atrial fibrillation, factor V deficiency, thrombophilia, among others. Approximately half of the participants (50.6%) had at least one chronic disease, allowing the performance of the bSens.INR System to be evaluated in a clinically diverse population.

The participants also used a wide variety of concomitant medications, including antihypertensive agents, diuretics, lipid-lowering drugs, antidiabetic agents, antiplatelet agents, anticonvulsants, antidepressants, antiretroviral drugs, and other long-term treatments. The most frequently used medications included metoprolol, simvastatin, enalapril, losartan, hydrochlorothiazide, furosemide, amlodipine, metformin, and omeprazole. Patients using acetylsalicylic acid, atorvastatin, dapagliflozin, paracetamol, diazepam, and several other medications were also evaluated.

Analysis of the bSens.INR System results that fell outside the acceptable limits calculated from the reference results did not demonstrate a statistically significant association between loss of accuracy and factors such as age, sex, presence of chronic disease, or overall medication use. However, some factors requiring attention as potential interfering factors were identified. These included hematological alterations, particularly low hematocrit values and marked changes in platelet counts. To further investigate the potential influence of hematocrit on measurement agreement, Graph 4 presents the Bland-Altman analysis with measurements from participants with low hematocrit highlighted in red. The potential influence of hematocrit on bSens.INR analytical performance was investigated using 173 paired determinations from 50 patients, with hematocrit values ranging from 31.8% to 52.3%. No statistically significant correlation was observed between hematocrit and measurement bias (Spearman's ρ = 0.102, p = 0.183). Similarly, when the analysis was performed using the mean bias for each patient to account for repeated measurements, no significant association was identified (Spearman's ρ = −0.039, p = 0.787). These findings suggest that hematocrit was not consistently associated with systematic measurement bias within the evaluated range. However, the potential influence of hematocrit on measurement agreement cannot be excluded, particularly at extreme values, and further investigations are needed to establish the robustness of the device across a broader range of clinical conditions.

**Graph 4:**
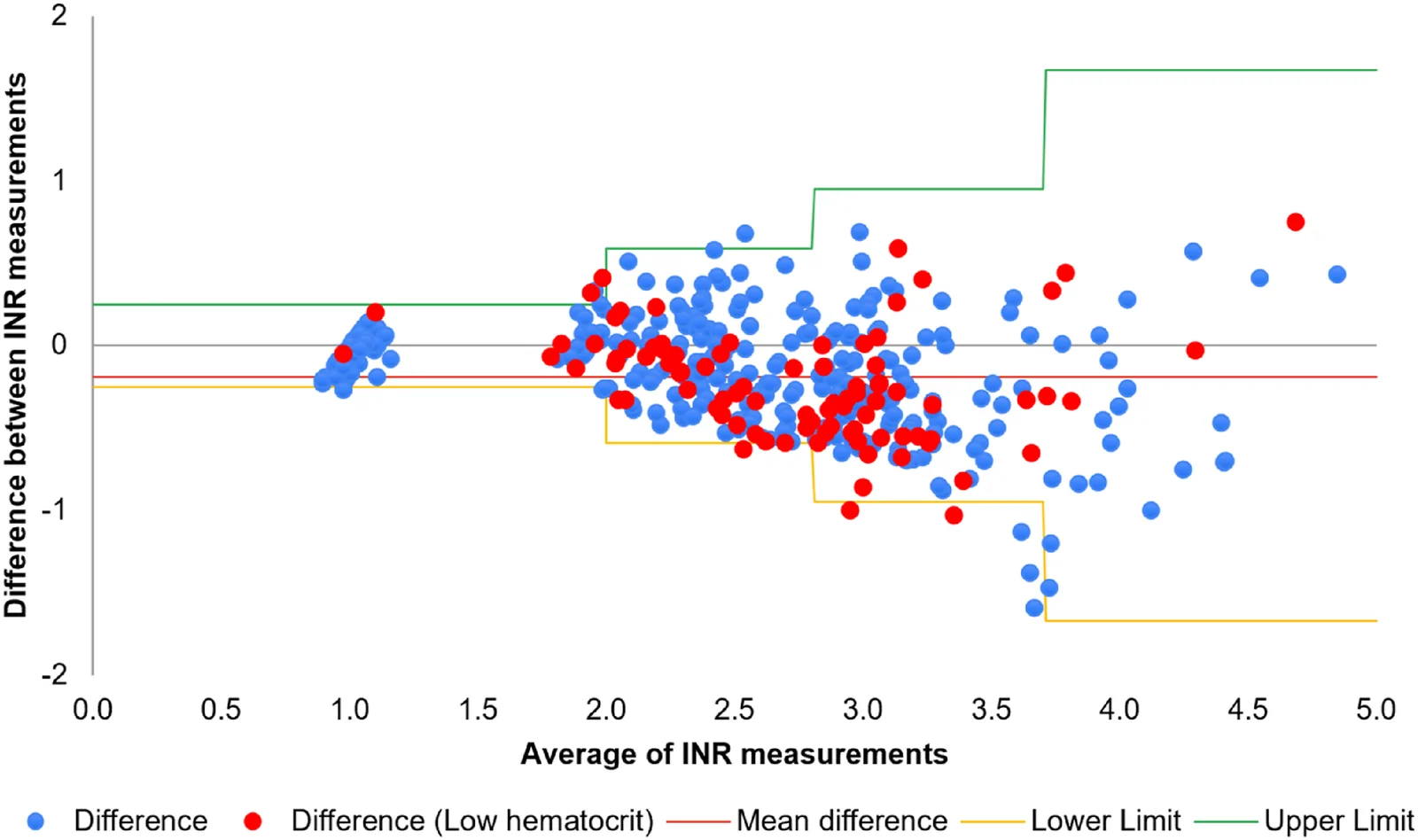
Bland-Altman analysis of the differences between the compared methods as a function of average INR values, considering the bias by INR range, and with measurements from participants with low hematocrit values highlighted in red.

Furosemide use also showed an exploratory signal of association with the occurrence of discordant results. Due to the small proportion of participants using this medication (9.1%) and the low number of rejected results (4.0%), this finding does not support classifying furosemide as a confirmed interfering factor. However, it may be considered a potential interfering factor that warrants further evaluation.

APS and the presence of lupus anticoagulant (LA) represent additional conditions requiring attention, as they have previously been reported as potential sources of interference in point-of-care testing. In this study, participants with APS accounted for 5 of the 77 participants evaluated (6.5%); however, some participants did not have a confirmed diagnosis and only reported suspected APS. The confirmed cases accounted for 9 duplicate determinations among the 200 duplicate tests performed (4.5%). Graph 5 presents the Bland–Altman analysis, highlighting in yellow the INR measurement differences obtained from participants with confirmed antiphospholipid syndrome (APS), while the remaining measurements are shown in blue. All measurements from participants with APS fell within the established limits of agreement between the bSens.INR device and the laboratory reference method. However, given the limited representation of participants with APS in the study population, these findings should be interpreted with caution. Further studies involving a larger number of patients with APS are warranted to more comprehensively investigate the potential influence of this condition on the analytical performance of the device.

**Graph 5:**
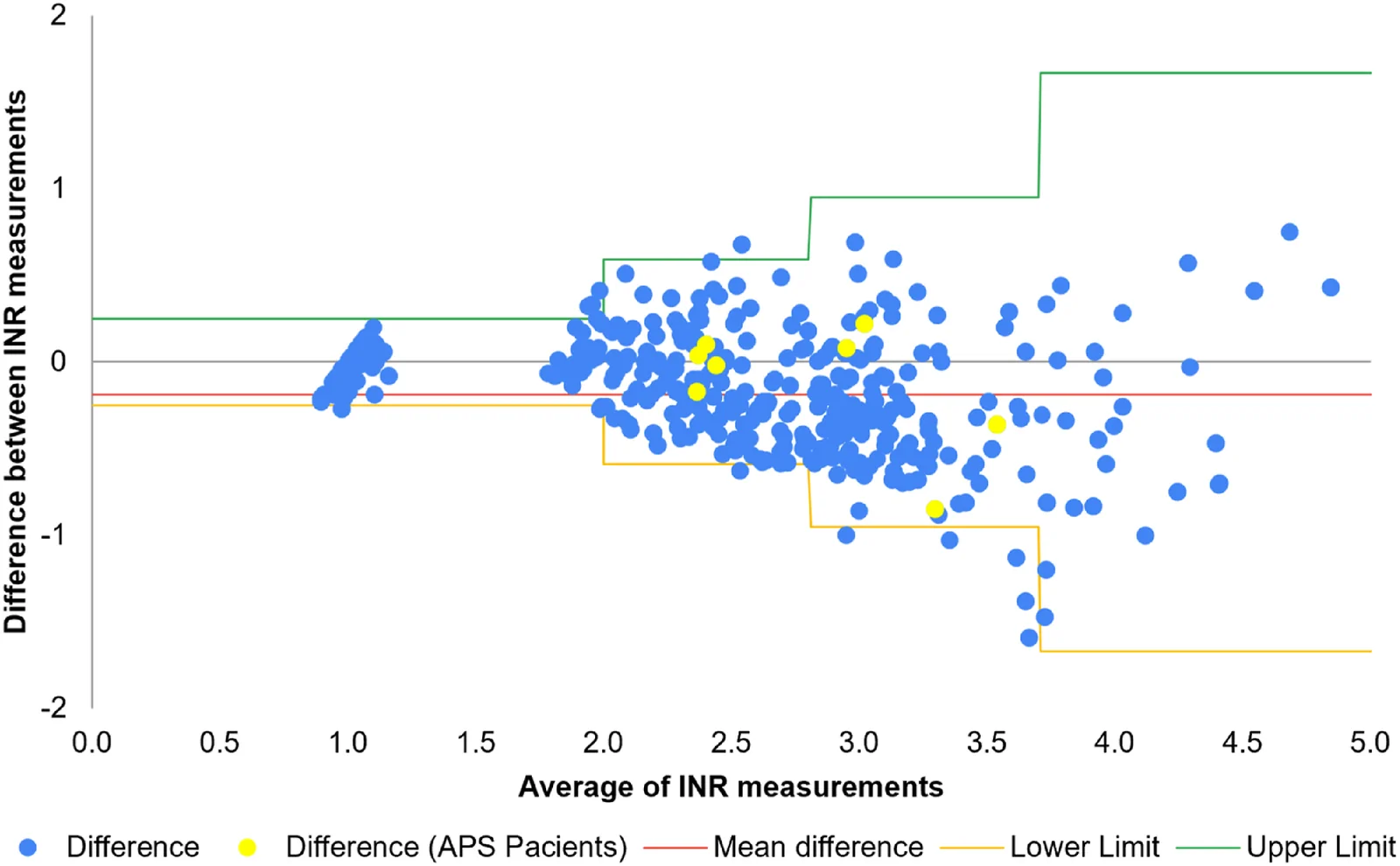
Bland-Altman analysis of the differences between the compared methods as a function of average INR values, considering the bias by INR range, and with measurements from participants with APS highlighted in yellow.

In patients with LA-positive APS, discrepancies between point-of-care (POC) INR and laboratory-based INR measurements have been reported. Noordermeer et al. (2023) [21] found disagreement exceeding the Clinical and Laboratory Standards Institute acceptance limits in 36% of patients when comparing POC-INR with INR measured using a coagulometer and the Owren method, which employs rabbit brain-derived thromboplastin; in 15% of patients when comparing POC-INR with Quick-INR, measured using the Quick method with a reagent containing recombinant human tissue factor; and in 21% of patients when comparing Quick-INR with Owren-INR. These discrepancies are clinically relevant because falsely elevated POC-INR values may lead to inappropriate reductions in VKA dosage, potentially increasing the risk of thrombotic events. In addition, high LA levels have been associated with cases in which coagulation times could not be determined using POC devices. One proposed explanation is that antiphospholipid antibodies interact with phospholipids in the thromboplastin reagent on the test strips, potentially altering coagulation reactions and resulting in inaccurate INR measurements. Other proposed mechanisms include antibody-mediated interactions with blood components that may affect clot detection [22]. Given these potential sources of interference and the limited number of participants with confirmed APS in the present study, the possibility of interference with the bSens.INR cannot be excluded and warrants further investigation.

Samples from individuals with different hematological and metabolic conditions were also evaluated, including variations in hemoglobin, hematocrit, and platelet counts. Overall, the clinical, laboratory, and pharmacological diversity of the study population did not result in a systematic loss of device performance. However, the findings related to furosemide use, hematological alterations, and APS should be considered potential signals of interference and warrant further investigation in specifically designed studies.

Therefore, points of attention regarding the potential interfering factors mentioned above were included in the bSens.INR System User Manual, advising users to interpret the results with caution and, when the result is inconsistent with the patient's clinical condition, to confirm the result using a reference laboratory method.

The overall summary of the evidence obtained throughout this study is presented in Table 8. Taken together, the results and evidence generated constitute the technical and scientific documentation included in the application dossier for registration of the bSens.INR with ANVISA, supporting the assessment of the product's performance, safety, and suitability for its intended use.

**Table 8:** Summary of clinical evidence.

Study	Protocol	Acceptance Criteria	Result
Accuracy	777 volunteers, with 200 duplicate determinations = 400 bSens.INR tests, using 10 strip lots and 5 devices, compared with the reference method and covering different INR ranges.	≥95% of results within the acceptance limits: INR <2.0: ±0.4 INR; INR 2.0-4.5: ±20% of the reference value.	197/200 duplicate results accepted (98.5%). Overall accuracy: 382/400 determinations (95.5%). Overall bias: −0.178 INR; r = 0.912. 94.5% of the 400 determinations were within the Bland–Altman limits of agreement.
Precision	60 volunteers, with 60 duplicate determinations = 120 bSens.INR tests, compared with the reference method and covering different INR ranges.	Difference between duplicates <10%.	60/60 duplicate results met the criterion (100%). INR <2.0: CV = 7.24%; INR 2.0-3.0: CV = 8.78%; INR 3.0-4.5: CV = 5.53%.
Intermediate Precision	3 volunteers, 5 days, 3 measuring devices, and 3 strip lots, totaling 90 tests, compared with the reference method and covering INR ranges <2.	No statistically significant differences by ANOVA (p > 0.05).	- ANOVA showed no significant differences associated with device, day, strip lot, or replicate (p > 0.05). Device: p = 0.300; Day: p = 0.734; Test strips: p = 0.315; Replicate: p = 0.629.
Analytical Sensitivity	400 bSens.INR tests compared with the reference method using linear regression and assessment of the applicability of the conventional concept of analytical sensitivity to bSens.INR.	Not applicable. bSens.INR does not directly quantify an analyte based on concentration; therefore, sensitivity determination using a conventional analytical calibration curve is not applicable. Performance is assessed based on agreement with the laboratory reference method, using analyses such as Bland-Altman to determine bias between methods.	Y = 0.8143X + 0.3072, where Y = INR measured by bSens.INR and X = INR measured by the gold-standard method. R^2^ = 0.8593; slope ≈ 0.8143; intercept ≈ 0.3072.
Analytical Specificity	Investigation of potential factors associated with discrepant results at INR >2.	Identification of factors potentially associated with INR deviations beyond the acceptable limits.	Potential factors of concern related to furosemide use, APS, and hematological parameters were identified and incorporated into the User Manual; however, they have not yet been established as causal sources of the observed deviations.
Measuring Range	Combined evaluation of accuracy, precision, intermediate precision, and stability studies, including samples close to and beyond the intended measurement limits.	Definition of a safe measuring range considering the observed performance and accuracy acceptance criteria.	- INR: 0.6-4.5; PT: 8-50 s.
Usability	80 duplicate self-tests performed by 40 volunteers and 80 duplicate determinations performed by a qualified healthcare professional = 320 determinations.	Difference between duplicates <10% for both lay users and healthcare professionals.	80/80 sets of four determinations met the criterion (100%). Lay users: CV = 4.71%; Healthcare professionals: CV = 6.27%.

The results obtained are considered sufficient to support the clinical safety and performance of the bSens.INR, as the studies were conducted using internationally recognized methodology and acceptance criteria established based on ISO 17593:2022 for PT/INR monitoring systems. The accuracy studies demonstrated adequate agreement between the bSens.INR and the reference laboratory method, supporting the reliability of the results across the established measurement range of INR 0.6 to 4.5 for oral anticoagulant therapy monitoring. Additionally, the repeatability studies showed variation of less than 10% between measurements performed under equivalent conditions, demonstrating the consistency of the system [13].

## 4 Discussion

Periodic PT/INR monitoring is essential for the management of patients receiving vitamin K antagonist therapy, particularly warfarin, due to its narrow therapeutic window and substantial interindividual variability in response. In this context, point-of-care devices represent an alternative to exclusively laboratory-based monitoring, allowing greater proximity between test performance and clinical decision-making. Studies involving self-monitoring and self-management have demonstrated that greater accessibility to testing may increase the frequency of INR measurements and promote the maintenance of patients within the therapeutic range [6], [7], [10], [23], [24], [25].

In this context, the clinical validation of the bSens.INR aimed to evaluate its performance not only in terms of agreement with the reference laboratory method, but also with regard to the consistency of results across repeated measurements, maintenance of performance under different testing conditions, and the influence of user-performed testing. Thus, the combined analysis of accuracy, precision, intermediate precision, and usability results provides a more comprehensive assessment of system performance and its suitability for the intended use.

Accuracy is one of the main parameters for evaluating POC systems intended for INR determination, as relevant differences from the laboratory method may lead to different interpretations of a patient's anticoagulation status. Previous studies have shown, however, that differences between POC systems and laboratory reference methods are expected and should be evaluated based on their magnitude and clinical relevance. Colella et al. (2012) [26], when comparing a portable device with a laboratory method in 200 duplicate determinations, observed good agreement between the methods, with a correlation coefficient of 0.91. Similarly, Bereznicki et al. (2007) [27] reported that only 5.1% of the results obtained with the CoaguChek XS showed differences greater than 15% compared with the laboratory method, with no results showing differences greater than 20% or discrepancies exceeding 0.5 INR units.

Fu et al. (2014) [28], using the recommendations of ISO 17593, also demonstrated comparable results between the CoaguChek XS Plus and the CA-1500 laboratory method. The authors observed a bias of −0.06 in the INR range of 2.0-4.5 and −0.01 across the entire evaluated range, in addition to differences of less than 0.5 INR units for 97% of samples with INR <2. These findings demonstrate that agreement assessment should consider not only the overall relationship between methods but also the magnitude of the differences observed across the different INR ranges. In this context, the results obtained for the bSens.INR, including a bias below 0.5 INR units, are consistent with the performance reported for POC technologies already used for this purpose [13].

It is also important to consider that the differences observed between the bSens.INR and the reference laboratory methods occurred despite substantial differences in their analytical methodologies. While the bSens.INR uses recombinant human tissue factor and electrochemical impedance measurements, the reference laboratory systems employ rabbit brain-derived thromboplastin and clot detection based on changes in the physical properties of the sample. Furthermore, the bSens.INR requires only approximately 8 μL of capillary whole blood obtained by fingerstick, whereas the laboratory methods use plasma obtained from venous blood samples. Therefore, the comparison involves not only different analytical detection principles but also differences in thromboplastin origin, sample type, and sample volume. Considering these methodological distinctions, the relatively small overall bias observed between the methods (−0.178 INR) is an encouraging finding, suggesting that the bSens.INR can provide results in reasonable agreement with laboratory-based measurements despite employing a substantially different analytical approach. Nevertheless, these methodological differences may contribute to the discrepancies observed, particularly at higher INR values, and further studies are needed to determine their individual effects on measurement agreement.

A particularly relevant finding observed during the validation of the bSens.INR was the reduction in accuracy at higher INR values, especially above 3.7. However, this behavior does not appear to be exclusive to the system evaluated. Kim et al. (2023) [29], in an analysis of 400 samples from patients receiving warfarin therapy, observed consistent results between the CoaguChek XS Plus and the ACL TOP 750 laboratory method within the therapeutic range, but an increasing tendency for differences in the supratherapeutic range. Similarly, Vazquez, Fleming, and Johnson (2017) [30] observed a reduction in the relative accuracy of POC devices at INR values above 3.0, requiring the application of a correction factor in that study to improve performance within this range.

These findings reinforce the importance of evaluating the performance of POC systems across different INR ranges rather than relying solely on a global measure of agreement. Despite the reduction in accuracy observed at INR values above 3.7, the bSens.INR demonstrated an overall accuracy of 98.5% across the 200 determinations evaluated, without the need for a correction factor. The occurrence of more pronounced differences at higher INR values, which has also been reported for commercially established systems, should be considered when interpreting results and establishing the conditions and limitations for use of the device [31].

In addition to accuracy, precision is essential to demonstrate that the system is capable of producing consistent results when the same condition is assessed repeatedly. In the present study, 100% of the duplicate tests met the established acceptance criteria for repeatability, demonstrating consistency among determinations performed under the same conditions. This finding complements the accuracy assessment, as it demonstrates that the observed performance depends not only on the agreement with the reference method but also on the system's ability to provide consistent results across repeated measurements.

Intermediate precision extends this assessment by introducing sources of variation that may be present under routine conditions, allowing evaluation of whether system performance is maintained in the presence of the variables considered in the experimental design. The absence of significant effects associated with the evaluated conditions reinforces the stability of the system in response to the variations introduced during the study. Thus, the repeatability and intermediate precision results, considered together with accuracy, provide complementary evidence supporting the reliability of the bSens.INR measurement process.

Another relevant aspect of a POC system is the influence of the user on the test result. Unlike laboratory methods performed exclusively by trained professionals, technologies intended for decentralized use must account for potential errors related to device handling, sample application, and execution of the steps described in the instructions for use. The literature indicates that the benefits associated with self-monitoring depend not only on the analytical performance of the device but also on adequate training, clear instructions, and quality control. In this context, the usability assessment of the bSens.INR complements the performance evidence by determining whether use by the intended user introduces relevant differences compared with results obtained by a qualified professional [32].

The population included in the validation study also allowed an exploratory assessment of potential factors that could influence system performance. Hematological parameters, clinical conditions, concomitant medications, and sample characteristics were considered, including hematocrit, platelet count, hemoglobin, the presence of antiphospholipid syndrome/lupus anticoagulant, chronic diseases, and medication use. However, since this assessment was performed descriptively and based on frequency analysis, without a specific controlled experimental design for interference testing, these results do not allow causal relationships to be established between these factors and any differences observed between the bSens.INR and the reference method. Therefore, the findings should be interpreted as exploratory and as supporting the identification of factors that may require further specific investigation [33], [34].

Overall, the literature demonstrates that POC systems for anticoagulation monitoring can achieve performance comparable to that of laboratory methods, although differences between methodologies may occur and become more pronounced within certain INR ranges. At the same time, clinical studies have shown that the availability of POC systems may promote more frequent monitoring and improved anticoagulation control, particularly when their use is supported by adequate training and professional follow-up. Therefore, the clinical relevance of a new POC technology should be assessed by considering its performance compared with the reference method, analytical consistency, and use under the intended conditions [11].

Taken together, the accuracy, precision, intermediate precision, and usability results obtained during the clinical validation of the bSens.INR, combined with the exploratory assessment of potential interfering factors and the available literature on established equivalent technologies, support the suitability of the system for its intended use and provide evidence of its performance and safety for PT/INR monitoring. The results also highlight the importance of continuous performance evaluation across different INR ranges, particularly at higher values, in order to appropriately establish the conditions and limitations for use of the system.

## 5 Conclusions

The present study provided clinical evidence supporting the adequacy of bSens.INR performance under the evaluated conditions and contributing to the characterization of its intended use as a point-of-care system for PT/INR monitoring. The data generated form part of the product's technical and scientific documentation and will be used to support the registration application process and regulatory evaluation by ANVISA.

As future perspectives, additional studies may expand the evaluation of system performance in specific populations and clinical conditions, as well as investigate, under controlled conditions, potential interfering factors identified during the validation, including hematological variations, clinical conditions, and concomitant medications. Increasing the number of samples in the higher INR ranges may also contribute to a more comprehensive characterization of system performance in this range.

Following completion of the regulatory process and potential authorization for commercialization, monitoring the performance of the bSens.INR under real-world conditions may provide additional evidence regarding its use in clinical practice. Future studies may also evaluate its application in home self-monitoring strategies and its integration with remote monitoring and telemedicine tools, thereby expanding the possibilities for decentralized monitoring of anticoagulant therapy.

## CRediT authorship contribution statement

**Julia Konzen Moreira:** Validation, Supervision, Methodology, Funding acquisition, Conceptualization. **Gabriela Victória de Mello Jantzch:** Validation, Methodology, Formal analysis. **Duane da Silva Moraes:** Writing – review & editing, Methodology. **Bruna Ferri Serafini:** Validation, Data curation. **Milleny Germann Souza:** Validation, Data curation. **Marcele Arais Hocevar:** Writing – original draft, Formal analysis. **Rodrigo Ritzel Bernasconi:** Software, Formal analysis, Data curation. **Fernanda Santos dos Santos:** Supervision, Formal analysis. **Willyan Hasenkamp Carreira:** Funding acquisition, Conceptualization.

## Declaration of generative AI and AI-assisted technologies in the writing process

During the preparation of this manuscript, the authors used ChatGPT (OpenAI) to assist with language editing, translation, and improvement of readability. The authors subsequently reviewed and edited the content and take full responsibility for the final version of the manuscript.

## Foundings

This study received financial support from the National Council for Scientific and Technological Development (CNPq), the Coordination for the Improvement of Higher Education Personnel (CAPES), and the Brazilian Funding Authority for Studies and Projects (FINEP). The authors gratefully acknowledge these institutions for the financial resources provided to support this research.

## **Declaration of competing interest**

The authors declare the following financial interests/personal relationships which may be considered as potential competing interests: Julia Konzen Moreira reports financial support was provided by Cooperative Academy of Professional Education. Gabriela Victoria de Mello Jantzch reports financial support was provided by National Council for Scientific and Technological Development. Willyan Hasenkamp Carreira reports was provided by Finep. Duane da Silva Moraes reports financial support was provided by Coordination for the improvement of Higher Education Personnel. Milleny Germann Souza reports financial support was provided by Coordination for the improvement of Higher Education Personnel. Bruna Ferri Serafini reports financial support was provided by Coordination for the improvement of Higher Education Personnel. Marcele Arais Hocevar reports financial support was provided by National Council for Scientific and Technological Development. Rodrigo Ritzel Bernasconi reports financial support was provided by National Council for Scientific and Technological Development. Fernanda Santos dos Santos reports financial support was provided by Coordination for the improvement of Higher Education Personnel. Julia Konzen Moreira reports a relationship with Biosens Desenvolvimento e Indústria de Biossensores Ltda that includes: consulting or advisory. Willyan Hansemkamp Carreira reports a relationship with Biosens Desenvolvimento e Indústria de Biossensores Ltda that includes: equity or stocks. Duane da Silva Moraes reports a relationship with Biosens Desenvolvimento e Indústria de Biossensores Ltda that includes: consulting or advisory. Gabriela Victoria de Mello Jantzch reports a relationship with Biosens Desenvolvimento e Indústria de Biossensores Ltda that includes: employment. Bruna Ferri Serafini reports a relationship with Biosens Desenvolvimento e Indústria de Biossensores Ltda that includes: employment. Milleny Germann Souza reports a relationship with Biosens Desenvolvimento e Indústria de Biossensores Ltda that includes: employment. Julia Konzen Moreira has patent BIOSSENSOR ELETROQUÍMICO, USO DO BIOSSENSOR ELETROQUÍMICO, MÉTODO PARA MEDIÇÃO DA COAGULAÇÃO SANGUÍNEA E EQUIPAMENTO ELETRÔNICO DE LEITURA DA COAGULAÇÃO SANGUÍNEA pending to BR10202600918. Most of the authors have professional relationships with Biosens Desenvolvimento e Indústria de Biossensores Ltda., the company responsible for the development of the bSens.INR system evaluated in this study. These relationships have been disclosed above. This study was conducted as part of the clinical validation of the bSens.INR system, and the resulting data are also intended to support the technical documentation submitted to the Brazilian Health Regulatory Agency (ANVISA). All study procedures and data collection were conducted in accordance with the applicable ethical and scientific requirements. The reference PT/INR measurements were independently performed by qualified clinical laboratories with no affiliation with Biosens. The reference laboratories did not have access to the bSens.INR results when performing the reference measurements, thereby maintaining independence between the investigational and reference methods. The authors declare these relationships and circumstances to ensure full transparency regarding the development and evaluation of the product. If there are other authors, they declare that they have no known competing financial interests or personal relationships that could have appeared to influence the work reported in this paper.

## Data Availability

Data will be made available on request.

## **Abbreviations**

PT: Prothrombin time
INR: International Normalized Ratio
POC: Point-of-care
ANVISA: Brazilian Health Regulatory Agency
CAGR: Compound Annual Growth Rate
WHO: World Health Organization
ASSURED: Affordable, Sensitive, Specific, User-friendly, Rapid and robust, Equipment-free and Deliverable to end-users
ISO: International Organization for Standardization
ANOVA: Analysis of Variance
SUS: Unified Health System
ICF: Informed Consent Form
RDC: Collegiate Board Resolution
SD: Standard deviation
UL: Upper Limit
LL: Lower Limit
CV: Coefficient of variation
APS: Aniphospholipid syndrome
GRAPH: Graphic
HIV: Human Immunodeficiency Virus
